# “We Were Just so Sad and Devastated”: NICU Nurses' Stories of Caring for Families With Substance‐Exposed Pregnancies

**DOI:** 10.1111/nin.12691

**Published:** 2024-12-12

**Authors:** Amber C. Welborn, Meredith R. Gringle, Tracy Nichols

**Affiliations:** ^1^ Department of Nursing Appalachian State University Boone North Carolina USA; ^2^ Department of Public Health North Carolina Wesleyan University Rocky Mount North Carolina USA; ^3^ Department of Community and Population Health LeHigh University Bethlehem Pennsylvania USA

**Keywords:** child custody, NICU nurse, substance‐exposed pregnancy, trauma

## Abstract

This secondary analysis re‐examined stories of caregiving told by NICU nurses in the southeast US through a trauma theory lens expanding on research surrounding substance‐exposed pregnancies. Narrative analysis identified distress‐related experiences of nurses related to child custody decisions and outcomes, suggesting traumatic stress within this caregiving dynamic. Four distinct story types and three themes were identified across 23 stories, highlighting similarities and differences and illustrating how distress and trauma were experienced and may be manifested in care practices. Study findings suggest cumulative and residual effects from traumatic experiences can impact NICU nurses' well‐being and care provision, in part by promoting anticipatory trauma. The role of relationship‐building with families, as part of family‐centered care, was also implicated in the intensity of distressful experiences and may increase nurses' vulnerability to trauma as well as create pathways to stigmatizing interactions. Incorporating principles of relational ethics into nurse practice guidelines, while recognizing how care work can influence and be influenced by the potential pathways from trauma to stigma, may provide valuable support for nurses. The application of such a framework could potentially reduce distressing and/or traumatic experiences for nurses caring for families with a substance‐exposed pregnancy.

## 1 Introduction

Rates of maternal opioid use quadrupled from 2010 to 2017 (Hirai et al. 2021) alongside significant increases in maternal stimulant use (Jones, Nakagawa, and Johnson 2021). In response, the incidence of Neonatal Abstinence Syndrome (NAS) has increased dramatically, with recent estimates showing a rate of 6.7 per 1000 hospital births (Strahan et al. 2020). Previous research examined the stigma associated with caring for infants and families of substance‐exposed pregnancies (SEP) in the Neonatal Intensive Care Unit (NICU) (Nichols et al. 2020) as well as detailed the experiences of NICU nurses (Fraser et al. 2007; Maguire et al. 2012; Reese et al. 2021). Studies examining NICU nurses' perspectives and experiences have deepened our understanding of the intrapersonal, interpersonal, and structural dynamics that can shape stigmatizing responses (Nichols et al. 2020; Welborn et al. 2023) as well as described tensions experienced by nurses between their personal feelings and beliefs and their moral obligations and commitments to family‐centered care (FCC) (Welborn, Lewallen, and Nichols 2022; Fraser et al. 2007; Maguire et al. 2012; Reese et al. 2021). Few studies, however, have applied a trauma lens to understand NICU nurses' experiences caring for infants/families with an SEP. In high‐stress work environments, it is not uncommon for NICU nurses to report distress and trauma from caring for fragile patients (Beck, Cusson, and Gable 2017). Less is known about the distress and trauma specifically associated with caring for the stigmatized population of infants and families who have experienced an SEP. This paper adds to the literature by directly examining trauma and distress expressed in NICU nurses' stories of caring for infants/families with an SEP.

## 2 Background

Perinatal opioid use and NAS have increased across the United States (US) since the late 1990s (AHRQ 2021; Hirai et al. 2021). Although more current evidence‐based practice guidelines encourage shifting care provision to less acute environments where parents may be more available (Whalen, Holmes, and Blythe 2019), many infants with symptoms of an SEP are admitted to NICUs for observation and/or medical support. Infants who are admitted to intensive care for symptoms associated with an SEP spend an average of 16 days in the NICU (Whalen, Holmes, and Blythe 2019). Infants with an SEP experience variable symptoms, which may not be related to type and quantity of substance exposure, but may exhibit significant neurological and gastrointestinal dysregulation contributing to feeding difficulties and inconsolability (Anbalagan and Mendez 2022). Due to child welfare and safety laws, differing by country and state, mothers with an SEP may be evaluated for their ability to provide a safe home environment for the infant before discharge from the hospital. Outcomes of the investigation may include alterations in child‐custody from the biological parent. As a part of this evaluation, the nurse may be encompassed within child‐custody discussions, as an observer of maternal engagement and behavior. Intersecting social disparities, needs of families, and perceived roles of the nurse as both caregiver and maternal observer can further increase the complexity of family care needs (Smith et al. 2018).

Trauma is a multifaceted construct, containing primary, secondary, vicarious, and anticipatory subcategories. Primary trauma occurs when an individual directly witnesses a traumatic event. Secondary trauma, which evolved from the concept of compassion fatigue, occurs when an individual is aware of traumatic events experienced by another person. This causes both an emotional response around the awareness as well as distress related to the desire to help the person who has directly experienced trauma (Figley 1997). Both compassion fatigue and secondary trauma may lead to outcomes such as anxiety, sleep difficulties, and emotional extremes (similar to PTSD). Vicarious trauma describes negative changes in beliefs and thoughts due to prolonged empathetic relationships with people who have experienced primary trauma (Kim et al. 2022). Anticipatory trauma results from exposure to primary and secondary trauma and describes the worry that a traumatizing event will occur (Armstrong and Carlson 2019).

A robust literature exists on nurses' traumatization through caregiving situations where patients experience pain, loss, tragedy and/or death (Berger et al. 2015). Secondary traumatic stress has been reported by nurses in a variety of maternal‐child specialties, including nurse midwives (Kim et al. 2022), pediatric nurses (Berger et al. 2015), and labor and delivery nurses (Nicholls et al. 2021). Caregiving settings with children experiencing chronic illness (Maytum, Heiman, and Garwick 2004; Meadors and Lamson 2008), abuse or neglect (Cain and Gautreaux 2022), or near end of life (Meadors and Lamson 2008) are ripe for nurses to experience secondary traumatic stress due to the medical complexity and emotional labor associated with these patient scenarios. Comparatively, infants with symptoms of an SEP are not traditionally included in these high‐acuity patient care categories because these infants are typically medically stable (Anbalagan and Mendez 2022). However, Fortney et al. (2020) found NICU nurses experienced increased levels of distress when they perceived higher levels of infant suffering and potential for poor quality of life, which is a common assumption of some caregivers about infants with an SEP (Recto et al. 2020). In addition, increased attachment to an infant and/or family with NAS has been found to magnify a traumatic experience (Kendall‐Tackett and Beck 2022).

In addition to experiencing secondary trauma, NICU nurses who care for mothers and infants with an SEP may be especially vulnerable to vicarious (Archibald and O'Curry 2020) and anticipatory trauma related to infant discharge because of assumptions regarding the maternal fitness of mothers who use substances during pregnancy (Terplan, Kennedy‐Hendricks, and Chisolm 2015). Nurses describe concern that mothers who did not consistently visit their infant in the NICU may not provide for the infant's caregiving and comfort needs at home after discharge (Fraser et al. 2007; Maguire et al. 2012; Romisher, Hill, and Cong 2018; Shaw et al. 2016; Whittaker et al. 2016). Additionally, nurses have expressed fear and mistrust in anticipation of an infant's discharge when someone with a substance use disorder will have access to the infant. Doubts about maternal fitness and concerns of safety (Welborn, Lewallen, and Nichols 2022) may taint the typical celebratory experience of infant discharge (Smith, Love, and Goyer 2022) with a sense of caution related to infant outcomes post‐discharge in the home environment. As fear is a key feature of trauma (Ellis and Zaretsky 2018), feelings of distress over a discharge perceived as unsafe and/or anticipation of negative consequences may give rise to anticipatory trauma.

### 2.1 Rationale and Aim of This Study

Data were collected as part of a larger Critical Incidence (CI) study (Welborn, Lewallen, and Nichols 2022) designed to examine the caregiving dynamic between NICU nurses and mothers with an SEP within the context of the southeast region of the United States, where disparities in healthcare and legal consequences for mothers who use substances in pregnancy are magnified (Angelotta and Appelbaum 2017; Hand, Short, and Abatemarco 2017; Patrick et al. 2015). The original data contained descriptions of caregiving incidents in the NICU related to infants and families with an SEP. Interview question focused on both positive and negative memorable caregiving experiences. Participants were not specifically asked about child‐custody‐related issues, however preliminary analysis identified experiences of distress were often expressed alongside discussions of child‐custody decisions and outcomes. Results of the original study suggested trauma may be present in this caregiving dynamic, warranting a secondary analysis to explore how distress and trauma were expressed in the previously collected stories. The aim of this study, therefore, is to explore NICU nurses' stories of child‐custody concerns surrounding infants and families with an SEP through a sensitizing lens of trauma.

## 3 Methods

### 3.1 Design

This secondary analysis used a narrative approach to examine NICU nurses' CI (Kemppainen 2000) stories about providing care to infants/families with an SEP. The original data set contained 52 descriptions of caregiving incidents in the NICU related to infants and families with an SEP. Unexpectedly, approximately half of the stories in the original study centered child‐custody as a primary CI. Current study analyses explored these child‐custody stories.

### 3.2 Participants

#### 3.2.1 Recruitment and Sampling Strategy

Registered nurses who were English‐speaking, had three or more years of NICU bedside experience, were currently working in a level II or higher NICU with an average of 20 h per week in the Southeast region of the US, and could attest to having cared for infants/families with an SEP were recruited to participate in the original CI study. Social media recruitment strategies included a digital flier in social media groups for NICU nurses with approximately 10,000 members and hashtag followers, yielding 16 interested participants. Additionally, snowball sampling from an interested participant successfully recruited one participant. Potential participants provided their contact information through a digital survey link, where the Principal Investigator (PI) (first author) contacted them to verify eligibility and attain informed consent. Eight individuals were either not eligible to participate due to geographical location or did not respond to scheduling emails. Participants and the PI had no prior relationship; however, participants were aware the PI had a clinical background as a NICU nurse. Recruitment and enrollment were simultaneous with initial analysis, concluding with event saturation, lasting approximately 6 weeks. No participants dropped out of the study after interviews were scheduled.

#### 3.2.2 Sample

The sample for the original study included nine participants with data gathered over 5 weeks in 2019. Participants all identified as women, reported over 5 years of bedside nursing experience, and averaged over a decade of specialty NICU nursing experience. Most participants resided and worked in North Carolina, with ages spanning 28−60 years. Two‐thirds had earned a BSN or higher. The data set for the current analysis contains 23 events. Participants reported 1−3 events. One participant from the original data set is not represented in the current study. This was the first participant to be interviewed, with the fewest number of stories reported. Her demographic data does not contain outlying characteristics.

#### 3.2.3 Procedures

Interviews were conducted using Critical Incident Technique (CIT), designed to elicit a story in a broad sense, then prompting participants to expand the story to include additional considerations such as environmental influences, timing, and subtle details that may be overlooked in preliminary storytelling. This technique provides the opportunity to uncover details that add depth and richness to the story, creating a more complete description of the event.

Participants selected a pseudonym, completed a demographic questionnaire, and met with the PI for a password‐secure recorded digital interview. Eight participants chose to be interviewed online through Webex, a password‐secure virtual platform, and one participant was interviewed in person at the participant's residence. One interview was conducted in two parts, with the rest as a single session. The PI, a former NICU RN with an MSN‐Nursing Education degree, conducted all participant interviews, as part of her doctoral work. At the time of the interviews, the PI had previous experience in qualitative methodology including conducting a pilot study for the original study. Only the PI and participant were present for each interview. The interview guide, pilot‐tested before the study, was designed to collect memorable and impactful stories of nurses' experiences caring for infants and families with a SEP.

Semi‐structured interviews lasted up to 2 h, with offered breaks throughout. Using best practices in CIT (Kemppainen 2000), the interview guide prompted memorable stories, requested elaboration using alternate perspectives, and included questions about participants' nursing practices and follow‐up questions to gain additional details. The PI wrote field notes during and after each interview. Interview recordings were transcribed through Temi, a secure online service, with manual verification by the PI to revise the transcripts for complete accuracy and note significant interview features such as tone of voice, changes in tone, and emotion. Transcripts were additionally cleaned for personally identifying information. Incidents were logged and categorized while examining the level of event saturation. Transcripts were not member checked.

### 3.3 Data Analysis

All three authors immersed themselves in the original 52 events to identify narratives where child‐custody was a primary consideration of the participant. Researcher positionality was acknowledged and integrated within the analytic process as a key part of establishing trustworthiness (Mullet 2018). Authors identified child‐custody‐related narratives individually, meeting to compare and resolve discrepancies. This process resulted in a final data set of 23 narratives centering child‐custody decisions and/or outcomes as a primary event.

Narrative analysis uses a holistic approach, allowing researchers to consider multiple aspects of a story as it was told (Riessman 2008). For this analysis, issues of plot (what was reported), tone (expressions of affect), and point of view (perspective from which the story was told) were considered. As data were originally collected using CIT, what made the event memorable and why the participant wanted to share that particular story was also examined. All authors contributed to coding and analyses, including immersion into and exploration of the data, and memoing. Trauma was the sensitizing lens brought to the analysis with the understanding that child‐custody issues were a prime location for experiences of primary and secondary trauma. Stories were read and re‐read multiple times by all authors individually. After each round of individual readings, authors would write memos depicting what they saw in the stories using the trauma lens. Story typologies emerged from the data as researchers coded, discussed, and analyzed transcripts. Individual and collaborative critical reflective practices of memo‐writing, discussion, and synthesis of analysis ensured rigor (Engward and Davis 2015). Through this ongoing practice, team members developed a cohesive agreement of an emerging typology enriched by the interdisciplinary perspective of the research team. The development of four final story types reflect analytic saturation. The typology centered the emotional experience of the participant, what made the incident memorable to the participant, and how the participant framed the story along with narrative plot points. After reaching consensus on the fit of each narrative within the typology, stories were re‐read within each type, memoed, and discussed to construct themes that cut across multiple story types.

## 4 Results

The final typology included: Unicorn Stories, Shattered Stories, Grappling Stories, and Weathered Stories. While each story fits into a single category, there are elements of stories that may share similarities with other types. It is also important to recognize that stories were centered and categorized, not individual participants. A critical finding was that participants described multiple experiences and told stories that spanned across the four story types within their interview (See Table 1). The distribution within the typology depicts a variety of coexisting experiences for nurses as compared to a developmental arc (e.g., moving from “unicorn” to “weathered” stories as nurses sense of burn out increases) or linking story type to a characteristic or attribute of the nurses or institution. In other words, the typology reflects intersecting nurse experiences and accompanying sense‐making. It would be reasonable for the typology to represent a developmental arc, moving from “unicorn” to “weathered” stories as burn out increases, or for story type to be dependent upon a characteristic or attribute of the nurse and/or the context of their training or institution. Instead the distribution within the typology depicts the variety of co‐existing stories nurses experience and process in their daily lives. Below is a brief description of each type, followed by an abbreviated sample story.

**Table 1:** Variation across stories.

Type	Participants	# Words	Unique	“I”	Bonding	Plot points
Unicorn	Sarah	656	√	√	√	Kept up with family; saw child thriving as a toddler
	Candace	1140	√	√	√	Became friends with adoptive parents; adopted own child
	Candace	885	√	√	√	Mother listens to advice and made considerable effort
	Amanda	586	√	√	√	Mother turned her life around; staff assumes good outcome
Shattered	Sarah	1570	—	—	—	Mother arrested and still got custody
	Candace	821	—	—	—	Found out baby died of SIDS in a hotel room
	Parker	1866	√	√	√	Mother gave infant methadone; still gets custody
	Amanda	614	—	√	√	Bonds with infant; baby dies of SIDS under grandmother's care
	Amanda	905	—	√	√	Mother released from jail; still gets custody
	Amanda	937	√	√	√	Has conflict with grandmother, who feels unsafe with her
Grappling	Elizabeth	493	—	√	—	Mother in denial of drug use; staff can't connect with her
	Sarah	769	—	—	—	Mother, active user, lived next door to grandmother with custody
	Candace	1060	—	√	—	Didn't trust mom alone with infant; next day mother acts out
	Haley	616	—	—	—	Infant discharged to family facing criminal charges
	Haley	509	—	√	—	Concerns over kin care placement; gets insight from 3rd party
	Felicia	1221	—	√	—	Parents are difficult; friend from out of state gets custody
	Felicia	635	—	√	—	Parents have no family support; father asks for paternity test
Weathered	Elizabeth	235	—	—	—	Mother wanted to pick 1 of her twins
	Candace	285	—	—	—	Baby died of SIDS in residential treatment program
	Parker	505	—	—	—	Housekeeping found needles behind couch after discharge
	Barbara	598	—	√	—	Mother admits taking cocaine because tired of pregnancy
	Haley	198	—	—	—	Grandmother gets custody but mom lives with her full time
				—	—	
	Amanda	294	—	—	—	Mother doesn't participate in care; doesn't get custody

### 4.1 Unicorn Stories

This type comprises four stories. Unicorn stories were memorable because they were exceedingly positive but exceptionally rare. In most cases, these experiences initially left the participant with hope. Descriptions of maternal fitness were prevalent, with explicit details on what the mother did “right” and/or her efforts in caring for her infant. Stories were often framed around bonding or relationship‐building with the mother. Unicorn stories frequently used “I” statements, indicating a personal involvement with the experiences, and were told with a warm tone. Nurses verbalized feeling very present and engaged during the experience and described the mothers similarly.

#### 4.1.1 Sample Story

*One particular story sticks out in my mind whose mother was very active in the baby's care, yet she knew the baby was dealing with some withdrawal symptoms and knew that ‘Yes, I did this in the pregnancy, this caused this with the baby.’ But yet she wasn't afraid to own that and she wasn't afraid to step in and to be part of that care. I felt like they owned everything that had happened and were willing to go in headfirst to make everything happen, in assistance with us. In other words, they didn't give us a hard time. They didn't fuss about every little thing. They weren't picky, blah, blah, blah, blah, blah…like that. Baby went home with parents. I want to say [baby] was there for a couple months may be. They went home with the parents, and we did see them. I will never forget. We saw them at one of the follow‐up reunions. And we were all amazed. The baby, she looked like she was thriving. She looked very healthy. She has siblings and when we saw her, the parents were very loving and kind and it was just like, "Ah! I helped, you know." I knew that the parents had done everything that we had asked them to do and participated in the care plan. And when they were discharged, not only [did she go] with the family home, but there was a grandparent situation that they lived right next door. So they had familiar support, and knowing that they were going home not only with mom and dad, but grandparents were next door if they needed help. So that was a very rewarding issue there in taking care of an NAS baby. The support is everything. It's been a while, a very long time since we've seen her, but she was a toddler the last time we saw her at one of the meetings and she was doing very well. She actually had a new sibling in the family and um, she was just running around, very happy child. Um, but she had had a kind of a tough stint in the NICU while she was there, but she was…it's like you'd never would've known anything had happened and she looked very healthy, and her parents looked very happy. And they stated she was very healthy. So just, just seeing that is worth $1 million, is worth everything*.

### 4.2 Shattered Stories

Seven stories comprise the shattered category. Shattered stories also conveyed strong emotions but were memorable because of their tragic components. This type included some of the longest stories in the data set and provided numerous, very specific details. Shattered stories frequently included words like “horrible,” “traumatic,” “devastated,” and “awful.” In comparison to other story types, participants experienced intense displays of emotion when retelling these stories. Most stories included both “I” and “we” statements, with the “we” referring to the NICU team, not the mother/family. This demonstrated the participant's active role in the situation, but also a shared, unit‐wide experience. Over half of Shattered stories centered the experience of bonding, either with the mother or the infant. The established bond seemed to expound the negative event further, resulting in an even more painful experience for the nurse.

#### 4.2.1 Sample Story

*I'd say my most memorable one, truly… the actual experience in the hospital was not super memorable, which is weird to say. It was the first child that I took care of by myself … I really enjoy[ed] taking care of that child in the hospital. Unfortunately, the part that made it so memorable was about 3 months after he was discharged, we found out that he had passed away of SIDS at home under some very sketchy circumstances. And…that has stuck with me. I just remember some of the things that happened while he was in the hospital. I enjoyed taking care of him. Grandmother was his Guardian. She loved him so much and took such good care of him and it was just a very unfortunate thing that happened, after he went home. When I was thinking back through, looking at your questions, that is definitely what sticks out in the most in my mind. It was just horrible. Like we all took care of him…he was there a long time. He had some other issues going on, but his withdrawal was long and drawn out. It was just when we all found out that was what had happened. We just were so sad and devastated. That's unfortunately my most memorable. There was no father involved. Mom had actually been in a treatment facility for quite some time, for majority of her pregnancy actually. And I think the pressure and the stress of having an infant in the NICU kind of caused her to, relapse a little more than she could cope with. She kind of was involved in the beginning and then not so much toward the end of the stay. I really don't remember much about her. I just really remember their grandmother being the one to see about him. And unfortunately, the grandmother was fairly elderly, and had some health issues of her own. It was not a great situation, but she definitely loved him and wanted to do her best for him. The fact that he was there so long, he kind of became the unit's baby. We would all tote him around because he was several months old by the time he left. He…wasn't sleeping all day anymore. He needed interaction and he just would be worn around her in rounds, just everybody just knew who he was and [would] stop by and talk to them and play with him and interact with him. I think that's probably what made it so memorable. The grandmother was not around a whole lot while he was in the hospital, and she did her best. She was taking care of her mother and her daughter. She had a lot going on at home too. I feel like our relationship was as good as it could have been for the amount of time she was able to be there*.

### 4.3 Grappling Stories

Six stories were identified as Grappling stories. In these stories, participants also expressed emotions, primarily frustration, over the situation. Frustration was often expressed toward the social service system, especially as it related to policies, procedures, and varying expectations. Participants often shared a disconnect between the child‐custody decision at discharge and their personal experiences of working with the family. This produced intense feelings of fear and anger. Frustration related to working with families was also described. Many stories emphasized the social conditions surrounding the mother/family and included the more sensational aspects of this care. Along with frustration, participants expressed confusion over how to effectively form a relationship with the mother/family due to a variety of perceived barriers including trust with the infant's mother. The stories conveyed a sense the participant was trying to understand and/or connect with families but did not feel they were successful. Concerns about post‐discharge infant care prevailed within these stories, with most situations perceived as not having a positive resolution. Several of the stories also include “we” statements, indicating these are stories and/or families shared across the unit.

#### 4.3.1 Sample Story

*Now with this particular baby, I don't remember there being a significant others. I just remember…there being a mother and she was also one who came in. I can't say that she was high every time she came in, but there were times when she came in and she was. She did not have a lot of support while she was in the unit. She most frequently came in by herself when she did…She was another one that you could tell was, like I said, there's a pattern I think with these moms that they overcompensate. They try to make us think that they're good moms and they are, they're good moms in their own right, but they have a problem. But they don't realize they do and they're not admitting it because they're not willing to take steps to help care for themselves. This particular mom also was erratic in her visits, her calls, her behavior. And she, actually in the end when the baby had gotten better, had not participated enough for us to feel comfortable sending the baby home with her. So they did get care for the baby, foster care, but it was familial care. They put the baby with the grandmother who lived right next door to the mother. And I'll have to admit, that upset a lot of us because all she had to do was walk next door and there she is with her baby again. And she had not gone rehab or done any kind of care or anything. There was a situation where we knew the mom was, the baby was going to be right back with the same type of environment and family and mom care. Unless grandma just wouldn't let mom have the baby and that I don't know (laughs). So going back to the same situations when the parents haven't done anything to make things better is extremely frustrating and it makes you scared for the safety. Just the fact that you're worried. …I'm going to say, me personally, I'm worried for the baby's safety. I'm worried that they're not going to be taken care of. They're not gonna be fed properly. They're not going to be clothed properly. Because how can a caregiver in that situation, who is still using very actively, focus on that small baby enough to give that baby the needs and take care of her, like they should? I don't know this the baby moved back in with mom, but what is very obvious is the fact that mom can just walk out our door and walk right over there. And you know, if you have a grandma that's willing to let mom take the baby back over to the next door, you don't know that. But it's a possibility. I think there needs to be some distance, in cases like that cause it made it too easy for mom to have access to baby again. It was very, very difficult to build a rapport with her and to integrate and involve her into care with the baby. There wasn't, I would have to say, a whole lot of relationship there even though it was tried. You try as hard as you do with the other ones, but when they're not there and you might rely on them to be there, but they don't show up, it's very hard to be able to trust*.

### 4.4 Weathered Stories

Six stories were categorized as Weathered stories. Weathered stories were told with an absence of emotional affect. Stories were conveyed in either a perfunctory manner or with an emphasis on the more sensational aspects of the situation. In direct opposition to Unicorn stories, Weathered stories were described as common occurrences. They were also some of the shortest stories with limited details provided. While there were some expressions of concern over safety or disagreement with the child‐custody decisions, there is little personal involvement expressed. Participants told these stories with a hardened tone and seemed somewhat withdrawn or removed from any intimacy with the described individuals. In some, the participant described only indirect contact with the mother/family, but the story is well known within the unit.

#### 4.4.1 Sample Story

*We, I, I had a mom who, as soon as she was no longer an inpatient herself, when she was discharged, she was only able to come up and see the babies like may be 10 min a day. Like she would come, she would hold the baby, the baby would start crying, she put the baby down, and say I have to go get coffee or something and then she just wouldn't come back that day. And that happened day after day after day or you know, there may be a couple of days where she didn't come. That would be what would happen. Um, or she needs to go outside and smoke a cigarette or she need to do something other than hold her crying baby. Yeah. No, it's probably the best scenario I can think of for that issue. No. I know for a fact that several nurses have, had tried to have conversations with her about what was going on and she never really would open up. She just kind of would continue to say the same thing, but we kind of all at some point realized that was just how it was going to be and, it's how it was until, um, and actually that child is not released to her child‐custody. I think her sister ended up taking that baby him, but she was still in, in his life. But, um, not, not the primary guardian. I mean it was, it was kind of a case of she just wasn't ever there long enough for the right people to get to the right place, you know? And so people had a lot of phone conversations with her, but not a lot of face to face, um, conversations*.

### 4.5 Themes

The narrative analysis identified three cross‐cutting themes. Aspects of each theme can be seen within each story type but how it is applied varies across types. The themes, Building Relationships, Story Ownership, and level of Uniqueness, are described below:

#### 4.5.1 Building Relationships

Aspects of relationship building were present in most stories but differed by story type. Unicorn stories, overall, did not focus on the relationship built with the family and specifically with the mother, but elements of the story pointed to a level of trust and communication that suggested a working relationship. The one exception was the only story of adoptive parents as caregivers. The relationship between the participant and the adoptive parents as it evolved into an ongoing friendship was the center of this story.

Shattered stories generally centered around a relationship that was built or was building with the mother/family or with the infant. In the sample story, the strong bond with the infant and the description of the grandmother suggested a relationship was built. In these stories, the relationship was often broken or disrupted by a traumatic event, including learning of the death of the infant. The break in relationship contributed to the tragic nature of the event.

Descriptions of relationships in Grappling stories were often framed around the difficulty of building a relationship. In the sample story, the nurse stated it was difficult to build rapport with the mother and that there were no other support people. Concern over the placement of the infant with the grandmother may have been heightened due to the lack of a relationship as well as the perception the mother was not engaging in care.

Finally, in the Weathered stories, relationships were downplayed and not the focus of the story. While there were descriptions of the mother/family's behavior, no examples were provided to indicate a relationship was built. In the sample story, for example, the mother was described as not being present enough for building a relationship.

#### 4.5.2 Story Ownership

Story types also differed by ownership of the story with the use of pronouns providing specificity about character positionality. Unicorn stories were all told from the position of the participant. The pronoun “I” was prominent in the storytelling and the examples provided were clearly experienced by the participant. This was also mostly true for Shattered stories. Shattered stories, however, often included infants that were in the unit for a longer period. In these cases, both “I” and “we” pronouns were used but the examples provided were still described as being experienced by the participant. Grappling and Weathered stories were more likely to use “we” pronouns and to include a greater mix of examples that were told to the participant rather than a direct experience. There were also more instances where the participant was unaware or did not share specific details of the situation. The Weathered stories, in particular, are told more as “unit stories;” stories that have been shared and passed down across the staff in the NICU.

#### 4.5.3 Uniqueness

The degree to which a story was considered rare or common also differentiated between the types. Unicorn stories were always expressed as extremely rare. What made them memorable was the fact they so rarely occurred, and the ending was positive. These are told as stories of hope, as in the sample story where the participant stated learning the infant grew into a thriving toddler “is worth $1 million, is worth everything.” Shattered stories were not explicitly called rare or unique, but the tragic events stand out because they did not happen with most cases. Tragedies, such as infant death, are not completely unexpected but still shocking to learn. Other stories in this type focus on a break in relationship with the mother/family and these are described as unusual. In Grappling and Weathered stories, participants described occurrences as “fairly common” and mothers/families referred to as “another one” as if they belonged to a group where experiences were similar.

## 5 Discussion

This study examined the stories of NICU nurses who cared for infants from families with an SEP that centered on infant child‐custody. Our preliminary analysis revealed almost half (23 out of 52) of the stories included child‐custody decisions as a major plot point, lending support to our premise that child‐custody decisions are critical for understanding NICU nurses' experiences of caring for infants/families with an SEP. By applying a trauma lens to these stories, we constructed a typology consisting of four separate story categories: Unicorn, Shattered, Grappling, and Weathered. Story types differed by the perceived uniqueness of the story, the level of bonding experienced by the nurse, and the point‐of‐view or perspective of the story. It is important to note specific plot points did not differentiate story types. Except for Unicorn stories, which represent stories of hope, both positive and negative events happened across types. Instead, the nurse's perception of the event and specifically what made the event memorable led to the event categorization. The clinical understanding of psychological trauma as both an objective event and subjective experience, with an acknowledgment that event details themselves are less important than the impact on the person who experienced it (Center for Substance Abuse Treatment 2014) underscored event categorization, creating typologies which better reflect the individual perceptions of the participants.

Analysis also revealed that story typology varied within participants, with none of the participants sharing stories within only one type but instead across multiple types. This variation emphasizes that participants' perspectives are multidimensional, representing mental, emotional, and relational spaces where many experiences and outcomes are possible, including trauma. This finding has implications for the cumulative and residual effects of traumatic experiences. Regardless of the type of trauma, it is likely that, over time, traumatic experiences influence subsequent caregiving experiences. Initial interpretation of Unicorn stories led us to believe these experiences, which read as hopeful, might lead to more empathetic views of families with an SEP and more positive interactions. However, as analysis continued, it was apparent that another interpretation was quite possible as experiences may occur concurrently and/or precede other contrasting experiences. It is possible that the timing of individual experiences relative to past or present unit‐shared experiences may significantly impact the perceptions of current and future traumatic experiences.

Findings also suggest important differences between story types. Bonding with the infant and/or family differentiated Unicorn and Shattered stories from Grappling and Weathered stories. Among stories that highlighted a bonded relationship, when outcomes were perceived as positive, the experience was considered rare and filled the participant with hope. When the outcome was perceived as negative, however, the events left the participant feeling deeply distressed, verbalizing a dreadful feeling toward future caregiving for families with an SEP. When the nurse perceived a bond with either the infant and/or the family, experiences and feelings were magnified. Given the relationship between bonding and care provision found in this study as well as others (Zander, Hutton, and King 2010), it is important to recognize that trauma and relationship building with mothers may be bidirectional, supporting the need for further interrogation.

### 5.1 When FCC Is Not Straightforward

FCC is considered best practice for NICU care (Gooding et al. 2011) emphasizing the importance of care provider and family‐member partnerships in patient care. NICU nurses implement FCC by educating and collaborating with families around infant care, explaining care procedures, and building strong relationships with mothers and other family members (Griffin 2006). Gooding et al. (2011) suggest that nurses work to ensure that NICU experiences are “as personal as possible” (p. 24) while Priyadarsini (2020) underscores the importance of trust as central to FCC and positive NICU experiences. For NICU nurses working with infants and families of SEPs, a highly stigmatized population, the emphasis on personalized, relational, and empathetic care may increase their vulnerability to trauma. In addition to their own potential internalized biases around maternal fitness and/or post‐discharge concerns, building and navigating a relationship with families who have experienced and anticipate stigmatizing interactions within a system that does not provide extra time or support for these practices can set them up for failure (Welborn, Lewallen, and Nichols 2022). This experience is distinct from many other NICU nurse experiences because instead of a marker of infant wellbeing, discharge is often perceived as a threat to infant health.

Current best practices of FCC in maternal‐child caregiving are guided by Relationship‐based Care Theory which stems from Watson's Theory of Caring (Koloroutis 2004), guiding nursing practice and contributing to the professional identity of nurses in many cases. FCC is viewed as part of the very fabric of maternal‐child care; created from the ethics and practice of the entire profession. Therefore, if a nurse doesn't fully engage in FCC, they may become viewed, or view themselves, as a violator of professional ethics and stigmatized as a “bad nurse” in need of intervention. We see this dynamic most evident in Weathered and Grappling stories.

The traditional patient population in a high‐level acuity NICU includes medically‐fragile infants due to prematurity and other health concerns, who may not survive or may likely experience long‐term health issues. While the positive outcomes related to relationship‐based care are evident, the emotional vulnerability and risk to the nurse may be overlooked due to the expectation of the nurse to consistently exhibit this kind of care. Primary trauma, as related to the care of medically‐fragile infants, is most recognized in NICU nurses, but secondary, vicarious, and anticipatory trauma is also possible (Beck, Cusson, and Gable 2017) and potentially not uncommon. This care environment, where trauma‐inducing experiences already exist and may mingle with stigmatizing views of maternal fitness and resultant fear around post‐discharge wellbeing, increases the required vulnerability of the nurse to fully engage in and practice FCC. Thus, as Thompson (2021) argues, trauma is not only an individual‐level reaction but instead reflective of interconnected systems of harm. The trauma described by the nurses in this study represents NICU care practices, particularly FCC, that fail to adequately recognize the limitations of individual responses (e.g., building strong relationships) to addressing and ultimately dismantling fear and stigma.

Relationship building processes vary by individual and structural factors, context, (Shah, Ahluwalia, and Spicer 2023) and particular interpersonal dynamics between nurses and patients. However, the emphasis on relationship‐building as best practice may exert a flattening effect on the possibilities of genuine connection within a relational space. Shah, Ahluwalia, and Spicer (2023) argue that all of these variabilities make relationships and relationship‐building far more “unpredictable” (p. 3) than is posited in Relationship‐based Care Theory. Put another way, relationships exist on a continuum; some promote/result in high‐quality care and others do not.

### 5.2 Ethical Considerations of Relationships as a Professional Obligation

Austin, Bergum, and Dossetor (2003) write that “[r]elational ethics is about being with, as well as being for, the other” (p. 46). High‐quality care provision, therefore, not only depends on authentic relationships between caregivers (both professional and lay, e.g., family members) and care recipients, but also on deep commitment to the processes of building relationships that weave together scientific expertize and deeply co‐constructed mutual respect borne out of empathy (Austin, Bergum, and Dossetor 2003). Relational ethics argues that “correct” care decisions are always subjective, a product of relationships forged carefully out of rich communication among caregivers and recipients.

Bergum (2002) situates both nurse and patient as givers and recipients of relationship within relational ethics, noting the centrality of “mutuality and mutual respect” (p. 11) that grows from an embrace of the “back‐and forth” (p.11) between nurses and patients. From a relational ethics perspective, care is not bestowed from giver to receiver but constructed within specific, always‐emerging relationships. These relationships must not just incorporate but be grounded in “staying open to the vulnerability” (Austin, Bergum, and Dossetor 2003, 51) of care recipients.

While individual relational vulnerability (e.g., between nurse and patient) is central to relational ethics of care, institutions also play a key role in ensuring the possibility of equitable, ethical, relational care through the creation and maintenance of the context of caregiving. Requiring institutions to “stay open to vulnerability” (Austin, Bergum, and Dossetor 2003, 51) through policies and culture which not only encourage individuals to openly express experiences of trauma but also ensure that this expression can occur without fear of punitive institutional responses may help to make trauma experiences more intelligible and better understood (Thompson 2021). Intentional institutional support, to solicit more genuine understanding of nurse experiences may lead to more robust, empathetic support for nurses who work with families with an SEP. More research is needed to explore how a relational ethics of care can offer support for nurses and other staff who work with this population as they work to co‐create strong, care‐full relationships. Additional research might also consider how distressing and traumatic experiences may undercut the vulnerability that is critical to building the mutuality central to relational ethics. Promoting the formation of these intensive relationships as the primary driver of high‐quality care in the absence of structural level support for and understanding of the potential impact on nurses' health and well‐being is not only ineffective but unethical.

Dynamics around responsibility and blame are writ‐large within this issue. Care‐provision approaches for families with an SEP increasingly recognize the centrality of stigma as a barrier to care‐engagement, moving away from a moral‐model of care and toward an understanding of substance use as chronic disease. Nurses are often on the front‐lines of this paradigm shift which may require not only personal shifts in perspective around the meaning of substance use, but also practice‐level changes that both take up and accommodate the specific needs of (building relationships with) families with an SEP. The responsibility of care for families with an SEP may be very different than for other families in the NICU and nurses may sense an individual‐level of responsibility for effective care that is often not supported without specific training in addiction medicine (Ellick et al. 2024) and without attention to the specific sense(s) of vulnerability of nurses who engage in this care.

In parallel to shifting “responsibility” for SEP away from individual mothers, a paradigm shift around NICU nurse care could help reconceptualize care‐provision for families with a SEP as *part of* instead of *the entire* answer to recognizing, addressing, and supporting families with a substance use concern. Just as mothers who use substances during pregnancy must not be individually blamed for what is ultimately a deeply complex issue, nurses who care for families with an SEP must not be held individually responsible for managing discursive, ethical, and practical issues and challenges that arise as part of care‐provision. This kind of blame, whether directed at mothers/families or at nurses, violates relational care ethics, and has no place in high‐quality care.

### 5.3 Toward an Ethical Balance of Care

Bedside nurses' perspective on their preferences and ability to engage in potentially‐traumatizing care provision is critical. Anticipatory trauma emerged as an important and unique aspect of caring for infants/families with an SEP. Recognizing that as nurses care for more than one family at time, and that past caregiving experiences frame context for new experiences, the complexity of caregiving over time is highlighted. Integrating principles of trauma‐informed care into structural support for caregivers may increase resilience and help nurses manage expectations of mothers with a SEP, thus facilitating more opportunities for relational care.

The discussion of these results does not argue against nurses building relationships with families but rather acknowledges that an ethical caregiving environment for families with an SEP requires structural support for self‐awareness of the nurse and careful consideration of the complexity of the mother and family. As literature supports that trauma can lead to burnout (Buckley et al. 2020; Sweigart 2017), and data show that nurses experience multiple and, potentially, conflicting caregiving experiences, sometimes simultaneously across patients, little is known about their synergistic impact on individuals or the team. Consistency and continuity of patient care must be carefully balanced with the nurse's ability to provide care in high‐stress, potentially‐traumatic situations. Nurses must receive support from others to meet the professional and ethical obligation of relational care in a way that engages the relational care ethic of mutuality to ensure that this work does not sacrifice their well‐being in the process.

### 5.4 Pathways to Stigma

Although this analysis did not directly address stigma, stigmatizing attitudes, beliefs, and behaviors were found in the data that have been published elsewhere (Authors). The application of a trauma lens allowed us to see potential pathways from trauma to stigma. These potential pathways were especially evident in grappling and weathered stories. Grappling stories illustrate the frustration and lack of agency nurses feel around both trying to build relationships with families with an SEP and trying to understand decision‐making processes within child welfare cases. Weathered stories describe nurses' experiences when they are not engaged with the families and/or the stories have become institutionalized within the unit as common lore. Weathered stories also represent a lack of hope and a sense that the story is all too common. Both story types include multiple instances where participants questioned and/or criticized maternal fitness. Copeland (2022) describes two paths to stigma that map onto experiences reflected in these two story types.

The first path is through disruption of role expectations. In our data, nurses described mothers who did not meet their expectations of how a mother should behave and engage in care with her infant. Copeland (2022) claims the disruption of expectations leads to the labeling of a patient as difficult and ultimately to what they describe as the “RN‐unpatient relationship,” arguing that this leads to care that is procedural but not supportive. This “RN‐unpatient relationship” is described in both the Grappling and Weathered stories. This process of labeling and then distancing from a person or a group are fundamental steps in the stigma process (Link and Phelan 2001).

The second path named by Copeland (2022) is moral disengagement, described as several cognitive mechanisms that help nurses resolve their stigmatizing behavior with their ethical code. Of particular relevance to our findings is the mechanism of diffusion of responsibility, occurring when multiple team members treat the patient in a similar fashion and/or share similar perceptions of the patient. We can see this in the Weathered stories as well as several of the Grappling stories when participants use “we” more than they use “I.” Throughout these narratives, there is the sense that the unit has reached an agreement about the mother/family. Attribution of blame, another cognitive mechanism described by Copeland (2022), allows nurses to justify the withholding of relational care because of a fault in the behavior of the mother/family. Attributions of blame are more visible in Grappling and Weathered stories, where a relationship has not been formed.

### 5.5 Limitations of the Current Study

Although recruitment was completed through social media channels without geographical limitations, most participants resided in North Carolina, which may reflect differences in the assumptions and experiences of the participants. Healthcare organization norms and practices within North Carolina could have influenced the participants' experiences, with potentially differing experiences from nurses in other parts of the United States. Additionally, the sample may reflect bias toward NICU nurses with more negatively perceived caregiving experiences than other NICU nurses, inspiring their interest in participation. Participants who responded to the online recruitment flier may have recently experienced a trauma and therefore more connected to or emotionally triggered by the phenomenon of SEPs, resulting in more emotionally charged reports. Other characteristics of self‐selected participants and influences of social media narratives, may be specific to nurses who engage in social media and differ from nurses who do not engage in social media.

By focusing on stories that centered child‐custody after discharge, we were able to identify elements of both anticipatory and secondary trauma associated with caregiving. However, we are limited in our interpretations of the role of trauma in care‐giving given the study's design. As a secondary analysis of previously collected data that focused on stigma, this analysis suggests the link between trauma and stigma but we did not design the study to explore trauma specifically. Trauma tends to be conceptualized as an individual experience, potentially obscuring the role of systems and structures in creating and perpetuating “traumatic” issues (Thompson 2021). This critique becomes especially important when linking trauma and stigma as current understandings of stigma recognize the centrality of social structure(s) (Sheehan, Nieweglowski, and Corrigan 2017). Likewise, trauma theory guided the analysis of this study, but we recognize other theories could have shaped this study as well, resulting in potentially differing results.

## 6 Conclusion

This study examined stories of nurses who care for infants and families with an SEP. Findings emphasize the variability in nurse‐family relationships, caregiving approaches, and practices. More importantly, how the relationship is experienced can vary widely, with some relationships resulting in nurse trauma while others conclude without distress. Nurses' sense of anticipatory and secondary trauma related to child‐custody decisions connect to potential pathways for the formation and/or the proliferation of stigma. Findings suggest that the professional expectation of relationship‐building between nurses and infants/families with an SEP must acknowledge and consider the caregiving dynamic which exists within the context of stigma and potential trauma experienced by both NICU nurses and mothers with an SEP. When nurses' trauma is unrecognized or ignored, symptoms of trauma may be borne out by the nurse in other ways, such as negative communication patterns (Beck, Cusson, and Gable 2017), and accumulate over time. While relationship‐building is a central component of FCC and of NICU care provision, a better understanding of the complexities and ethics of relationship‐building is required to support nurses and prevent negative outcomes to patients, families, and nurses.

## Ethics Statement

All participants provided written informed consent before enrollment in the study.

## Conflicts of Interest

The authors declare no conflicts of interest.

## Data Availability

The authors have nothing to report.
